# Molecular Analysis of Activation-Induced Cytidine Deaminase Gene in Immunoglobulin-E Deficient Patients

**DOI:** 10.1155/2008/146715

**Published:** 2009-02-25

**Authors:** Sergio Roa, Maria Isidoro-Garcia, Ignacio Davila, Elena Laffond, Felix Lorente, Rogelio Gonzalez-Sarmiento

**Affiliations:** ^1^Molecular Medicine Unit, Department of Medicine, University of Salamanca, 37007 Salamanca, Spain; ^2^Department of Cell Biology, Albert Einstein College of Medicine, Bronx, NY 10461, USA; ^3^Department of Allergy, University Hospital of Salamanca, 37007 Salamanca, Spain; ^4^Centro de Investigación del Cáncer (CIC-IBMCC), University of Salamanca, 37007 Salamanca, Spain

## Abstract

Understanding how class switch recombination (CSR) is regulated to produce immunoglobulin E (IgE) has become fundamental because of the dramatic increase in the prevalence of IgE-mediated hypersensitivity reactions. CSR requires the induction of the enzyme AICDA in B cells. Mutations in AICDA have been linked to Hyper-IgM syndrome (HIGM2), which shows absence of switching to IgE as well as to IgG and IgA. Although isolated IgE deficiency is a rare entity, here we show some individuals with normal serum IgM, IgG, and IgA levels that had undetectable total serum IgE levels. We have analyzed the *AICDA* gene in these individuals to determine if there are mutations in AICDA that could lead to selective IgE deficiency. Conformational sensitive gel electrophoresis (CSGE) and sequencing analysis of *AICDA* coding sequences demonstrated sequence heterogeneity due to 5923A/G and 7888C/T polymorphisms, but did not reveal any novel mutation that might explain the selective IgE deficit.

## 1. INTRODUCTION

The prevalence of immunoglobulin-E-(IgE-) mediated hypersensitivity reactions, such as allergic asthma, rhinitis, hay fever, or food allergy, has been dramatically increasing for the last decades [1]. Total serum IgE levels tend to be higher in allergic patients compared with nonallergic individuals, although the diagnostic value of total serum IgE is limited [2] and the presence of specific IgE is not always equal to disease [3, 4]. The effectiveness of humoral immune responses depends on the capacity of B-cells to class switch from IgM to the other downstream isotypes. Class switch recombination (CSR) is a recombinational process that requires the introduction of double-stranded DNA breaks into the donor *μ* switch region, that is 5′ to the *μ* constant regions, and into a recipient *γ*, *ε*, or *α* switch region that is 5′ to each of those constant regions. The antibody repertoire is shaped not only by CSR, but also by somatic hypermutation (SHM) to create higher affinity antibodies. Both processes occur in centroblast B cells in the germinal centers of secondary lymphoid organs [5, 6]. A major break through in the understanding of how these processes are initiated was provided by the discovery of the mutagenic enzyme activation-induced cytidine deaminase (AICDA, also known as AID) [7–9].

Class switching to IgE requires two signals: the first is delivered by IL-4 and IL-13, and the second is provided by the interaction of the B-cell surface antigen CD40 with its ligand CD154 (CD40L) [10], which is transiently expressed on activated T cells and synergizes with IL-4 to induce AICDA-encoding messenger RNA and AICDA protein [11]. When initiating IgE switching, IL-4 induces the binding of STAT6 to a site in the 5′ region of the *AICDA* gene, and CD40 activation induces the binding of NF-kB to two sites in the same region of the *AICDA* gene [12]. Synergy between IL-4 and CD40 might be required to achieve a threshold level of *AICDA* expression for CSR to IgE [13]. Several groups have reported an association between serum IgE levels, allergic disorders, and polymorphisms in the *AICDA* gene [14–16], although this association is not completely understood and might vary among populations [17, 18].

Defects in CSR have been described in hyperimmunoglobulin M (IgM) syndromes, which are primary immunodeficiencies characterized by normal or elevated serum IgM levels with the absence of other isotypes [19]. A group of patients with the autosomal recessive form of the hyper-IgM syndrome (HIGM2) are known to have mutations in the *AICDA* gene [7, 20]. Since some of these mutations in AICDA are not in its active site, it has been assumed that they related to the targeting of AICDA. This is born out by the fact that mutations and deletions in the C-terminal region of AICDA result in the loss of class switching while SHM persists [21, 22], whereas mutations in the N-terminal part of AICDA lead to the loss of SHM and retention of CSR [23]. This suggests that there may be AICDA associated proteins that are required for the targeting to switch regions and raises the possibility that different proteins associate with AICDA to target it to each of the switch regions. One way to screen for such interactions is to search for mutations in *AICDA* that lead to selective CSR impairment in clinical groups with specific immunodeficiencies.

Isolated IgE deficiency is a rare entity and its association to clinical relevant immunodeficiency is controversial [24–29]. As opposed to hyper-IgM syndromes, the levels of other isotypes are normal in individuals with isolated IgE deficiency, suggesting the possibility of a selective CSR defect to this isotype. In the present study, we had the opportunity to investigate a rare group of 9 individuals with isolated IgE deficiency. In an attempt to further understand the contributions of AICDA to the mechanisms underlying CSR and IgE production, we performed a molecular characterization of *AICDA* gene in these subjects, to assess whether specific defects in AICDA are related to isolated deficiency of total serum IgE levels.

## 2. MATERIALS AND METHODS

### 2.1. Subjects

This study was performed in 9 patients with serum IgE levels below 2 kU/1 selected from a total of 643 patients that were referred during two consecutive years to the Allergy Department of the University Hospital of Salamanca (Spain) for an allergic evaluation. All of them gave informed written consent and the study was performed following the recommendations of the Ethical Committee of the University Hospital of Salamanca. Total serum IgE levels were measured in all patients by fluorescent enzyme immunoassay (ImmunoCAP total IgE, Phadia, Uppsala, Sweden), which exhibits a measuring range for undiluted serum of 2–5000 kU/l. Since IgE levels vary greatly in patients younger than 15 years, only patients older than 18 years were selected. IgE deficiency was defined as a total serum IgE level below 2 kU/l. Skin prick testing was performed following the European Academy of Allergology and Clinical Immunology (EAACI) recommendations [30] with a battery of common aeroallergens that included *D. pteronisynuss*, *D. farinae*, *L. destructor*, *T. putrescentiae*, *A. siro*, *G. domesticus*, *E. maynei*, mix of grasses, mix of trees, *P. judaica*, *C. album*, *A. vulgaris*, *P. lanceolata*, *O. europaea*, *A. alternata*, *C. herbarum*, *P. notatum*, *A. fumigatus*, dog, cat, hamster, horse, rabbit dander, and cockroach (ALK-Abelló, Madrid, Spain). Histamine (10 mg/mL) was used as positive control and saline was used as negative control. Before skin testing, antihistamines were discontinued according to published guidelines. Skin tests were considered positive if at least one wheal reaction of more than 3 mm of diameter after subtraction of the negative control was observed. IgM, IgG, and IgA levels were measured in all patients with IgE level below 2 kU/l, by nephelometry (Dade Behring Inc, Deerfield, Ill, USA).

### 2.2. DNA extraction, PCR primers design, and amplification reactions

Genomic DNA was extracted from peripheral blood lymphocytes using a standard phenol-chloroform protocol. Exons 1–5 and intronic flanking sequences of the *AICDA* gene were amplified using polymerase chain reaction (PCR). 3′ ends of all primers were designed to be located at least 45 base pair (bp) away from the splice junctions or the stop codon (Table 1). Amplifications were performed using 250 ng of DNA template, 1 *μ*L of each 10 *μ*M primer and 21 *μ*L of 2xPCR Master Mix (Promega, Madison, Wis, USA) in a final volume of 25 *μ*L in an GeneAmp PCR System9700 automated thermocycler (PE Applied Biosystems, Foster City, Calif, USA) with the same conditions: 4 minutes of denaturation at 94°C, followed by 30 cycles with a denaturation step of 1 minute at 94°C, an annealing step of 1 minute at 55°C, an extension step of 1 minute at 72°C, and a final 10 minutes extension at 72°C. Amplification of the samples was checked by electrophoresis in 2% agarose gels.

### 2.3. Mutation detection by CSGE (conformational sensitive gel electrophoresis)

Mutation detection enhancement (MDE) gels (Cambrex, Rockland, Me, USA) were used to analyze PCR products for the presence of mutations in the *AICDA* gene. 10 *μ*L of the PCR product were combined with 2 *μ*L of 6xTriple dye loading buffer (Cambrex, Rockland, Me, USA). The samples were heated at 95°C for 5 minutes, slowly cooled to 37°C and run on 0.5xMDE gels containing 0.6xTris-Borate-EDTA (TBE) buffer (1xTBE = 0.089 M Tris-Borate, 0.002 M EDTA, and pH 8.3). Gels were run overnight in 0.6xTBE at 20 V/cm for 12–18 hours, depending on the size of the PCR product. MDE gels were stained using PlusOne DNA silver staining kit (Amersham Biosciences, Uppsala, Sweden) according to the manufacturer's instructions. Heteroduplex bands were visualized on a transilluminator and documented.

### 2.4. Sequencing

Whenever a heteroduplex was detected, nucleotide sequence information was determined by direct sequencing of the PCR products. The genotyping of intron 2 (5923A/G) and exon 4 (7888C/T) polymorphisms were performed by direct sequencing in all cases. The same primers designed for PCR amplification were used for the sequencing analysis of the PCR products (Table 1). PCRs fragments were purified with MicroSpin S-300 HR Columns (Amersham Biosciences, Piscataway, Nj, USA) and sequenced using d-Rhodamine Dye terminator cycle sequencing kit and analysed with ABI Prism 377 genetic analyser (Applied Biosystems, Inc).

## 3. RESULTS

From a total of 643 patients that underwent determination of total serum IgE, 9 patients had IgE levels below 2 kU/l (1.4%). Strikingly, total serum IgM, IgG, and IgA levels were normal in all of them (Table 2). None of these patients had personal history of allergy or atopy and all of them had negative skin tests. No previous personal or familial history suggesting immunodeficiency was recorded in any of them. Nevertheless, an increase in nonallergic reactive airway diseases (i.e., nonallergic asthma) and autoimmunity (i.e., hyperthyroidism and dermatomyositis) was observed.

**Figure 1:**
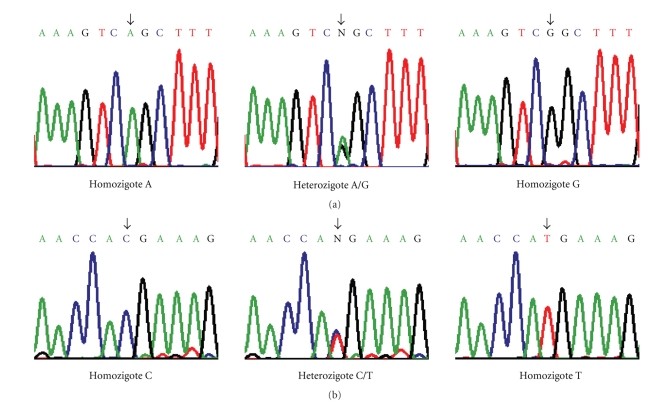
Sequencing analysis of regions 2 and 4 from human *AICDA* gene, where genetic variants were detected by heteroduplex. Panels show sequences of the different alleles corresponding to 5923A/G polymorphism at the 3′-flanking region of (a) exon 2 and 7888C/T polymorphism at (b) exon 4 of the *AICDA* gene. Arrows indicate the polymorphic sites.

MDE gel electrophoresis of PCR products spanning exons 1, 3, and 5 from *AICDA* gene revealed germ-line configurations. As expected, due to 5923A/G and 7888C/T polymorphisms [16], several heteroduplex patterns were detected in PCR products spanning exons 2 and 4, respectively. Sequence analysis of both regions in all patients identified different alleles for the polymorphic sites (Figure 1) but did not reveal any novel mutation.

Distributions of genotypes of the 5923A/G and 7888C/T polymorphisms were consistent with Hardy-Weinberg equilibrium. The distribution of genotypes of these polymorphisms in the 9 patients is shown in Table 3. We have studied a small number of patients because of the low prevalence of IgE deficiency. Although this is an important limitation to the study of this condition, the genotype frequencies of the synonymous 7888C/T polymorphism at exon 4 (Reference SNP Identification rs2028373 [31]) did not deviate from expected values previously reported [16, 18]. In the case of the *AICDA* intronic 5923A/G polymorphism, genotype frequencies were similar to those reported in NCBI single nucleotide polymorphism database, according to Reference SNP Identification rs2518144 [31]. The diplotype distribution of both polymorphisms was heterogeneous. No specific haplotype was detected in this population.

## 4. DISCUSSION

The level of serum IgE is usually 10,000 to 50,000-fold lower than the level of serum IgG and its production is tightly regulated. The overall role of IgE in immunity is not completely understood, but it seems to be involved in the defence against parasitic infections. Reports about IgE deficiency are limited and their association with clinically relevant immunodeficiency is still controversial [24–29]. We have evaluated the clinical characteristics of a group of patients with serum IgE levels below 2 kU/l, but normal IgM, IgG, and IgA serum levels (Table 2). In our setting of an outpatient allergy clinic, only 9 out of 643 (1.4%) patients that underwent IgE determination had serum total IgE levels under the detection threshold (2 kU/l). There is limited information about prevalence of IgE deficits. Levy et al. [32] found an incidence of 16.5%, but their threshold was 10 kU/l, and Smith et al. [29] found 10.5% of patients with IgE levels below 2.5 kU/l. Our incidence is lower probably due to the lower threshold that we used. Although controversial, there are some reports on local IgE production [33] and therefore we cannot absolutely discard the possibility of local CSR to IgE in tissue. Nevertheless we believed that this fact is highly improbable because it has been shown that patients with local production of specific IgE did not have undetectable total IgE levels and they suffered from perennial rhinitis, usually with seasonal symptoms [34].

None of the patients with IgE deficiency included in our series showed personal or familial clinical data of immunodeficiency but three patients presented nonallergic asthma, and autoimmune disorders were present in two patients. This is in agreement with the study of Smith et al. [29], which found a higher prevalence of both conditions in patients with serum IgE levels below 2.5 kU/l, suggesting the existence of common genetic factors that may predispose to both IgE deficiency and autoimmunity. Other authors have proposed that deregulation of molecules and signals that play a key role in B-cell activation and terminal differentiation could be involved in initiating or maintaining autoimmunity [35].

AICDA plays an essential role in CSR, which is impaired in both AICDA-deficient mice [8] and in patients with HIGM2 syndrome [7, 20]. An association between serum IgE levels and AICDA has been previously reported, and several studies have shown the association between polymorphisms in the *AICDA* gene and allergic disorders [14–16], although this association is not completely understood and might vary among populations [17, 18]. Serum IgE levels have an inherited non-Mendelian component, although there is one report that showed an autosomal dominant pattern with a variable degree of penetrance in patients with IgE deficiency [28]. Exploration of genetic regulation in IgE responses, based on both candidate gene approaches and linkage studies has led to the report of several sets of molecular and cellular interactions that are essential for IgE synthesis [36]. Nevertheless, no study of susceptibility loci has been performed on patients with undetectable serum IgE levels. Here, we have examined whether mutations in the *AICDA* gene might explain the phenotype of our cohort of patients with isolated IgE deficiency and affect the specificity of switching towards IgE production. We have identified two previously described polymorphisms (intron 2 5923A/G and exon 4 7888C/T) in the *AICDA* gene, but no defects in the *AICDA* coding or flanking regions account for the IgE deficit in our cohort. Previous reports and public databases on these polymorphisms did not show any evident association with *AICDA* expression levels, splicing or function. We cannot rule out that mutations in the promoter region, epigenetic changes or posttranslational modifications in AICDA could be involved in the decrease of IgE levels. In addition, other factors involved in immunoglobulin class switching besides AICDA might be implicated. The characterization of such factors would be important in understanding the specific targeting of AICDA to the *ε * switch region and the molecular basis of isolated IgE deficiency.

## 5. CONCLUSIONS

The exploration of various types of clinical immunodeficiencies provides a unique opportunity to better understand the molecular basis of immunoglobulin class switch recombination. Here, we evaluated the clinical characteristics of a cohort of patients with isolated IgE deficiency, which exhibited a higher prevalence of nonallergic asthma and autoimmunity disorders. We analyzed *AICDA*, the master gene required for CSR, in these patients to see if there were specific mutations that would result in impaired CSR and explain their undetectable serum IgE levels. Two previously described polymorphisms (intron 2 5923A/G and exon 4 7888C/T), but no mutation, were detected in *AICDA* gene. Further search for alternative causes of isolated IgE deficiency could open new ways to understand specific CSR and IgE production.

## Figures and Tables

**Table 1:** *AICDA* primers, PCR conditions, and size of the fragments used in the study.

Exon	Primer designation	Primer sequence	Annealing		Product size (bp)
			Temperature (°C)	Position*	
1	hAID1F	5′-GCCGTTGGGGTACCTGGTGG-3′	55	−179	280
	hAID1R	5′-ATGAGAGAAAGGGATAGCTA-3′		+100	
2	hAID2F	5′-AGCCCAAGTAATGACTTCCTTA-3′	55	+5661	321
	hAID2R	5′-ACCATCAGCAGGTGGCTCTAA-3′		+5981	
3	hAID3F	5′-GACTAAGGCTACCAGAGCCG-3′	55	+7221	440
	hAID3R	5′-GCCCACTTCTTCCCCTCGAG-3′		+7660	
4	hAID4F	5′-GTGAATGGCTCAGAGACAAGG-3′	55	+7716	364
	hAID4R	5′-ATCAGATGAAAACTGAGAGTGA-3′		+8079	
5	hAID5F	5′-GTTACAAAGCCATCCACTCAG-3′	55	+8334	237
	hAID5R	5′-GAGAAGACTTGAAGGACTG-3′		+8570	

*Position of primers is according to GENBANK sequence Accession Number AB040430.1.

**Table 2:** Phenotype characteristics of patients with IgE hypogammaglobulinemia.

No.	Sex/Age	Clinical diagnosis	IgE	IgG	IgA	IgM	History of immunodeficiency/
			(kU/l)	(mg/dl)	(mg/dl)	(mg/dl)	autoimmunity
1	F/57	Normal	<2	1300	1822	147	No/No
2	F/37	Non allergic asthma	<2	735	156	226	No/No
3	F/65	Normal	<2	954	221	63	No/No
4	F/28	Contact dermatitis	<2	700	108	157	No/No
5	F/57	Normal	<2	1210	245	84	No/hyperthyroidism
6	M/44	Chronic urticaria	<2	859	257	69	No/No
7	F/59	Non allergic asthma	<2	1080	242	73	No/No
8	M/48	Non allergic asthma	<2	809	200	58	No/No
9	F/57	Normal	<2	1540	353	234	No/dermatomyositis

F: female, M: male.

**Table 3:** Genotype distribution of 5923A/G and 7888C/T polymorphisms from *AICDA* gene in our population.

Patient	5923A/G	7888C/T
1	GG	TT
2	AG	CT
3	AG	TT
4	GG	CT
5	GG	TT
6	AA	CT
7	AG	CC
8	GG	CT
9	GG	TT

## References

[1] Poulsen LK, Hummelshoj L (2007). Triggers of IgE class switching and allergy development. *Annals of Medicine*.

[2] Klink M, Cline MG, Halonen M, Burrows B (1990). Problems in defining normal limits for serum IgE. *Journal of Allergy and Clinical Immunology*.

[3] Bodtger U, Poulsen LK, Malling H-J (2003). Asymptomatic skin sensitization to birch predicts later development of birch pollen allergy in adults: a 3-year follow-up study. *Journal of Allergy and Clinical Immunology*.

[4] Bodtger U (2004). Prognostic value of asymptomatic skin sensitization to aeroallergens. *Current Opinion in Allergy & Clinical Immunology*.

[5] Durandy A (2003). Activation-induced cytidine deaminase: a dual role in class-switch recombination and somatic hypermutation. *European Journal of Immunology*.

[6] Liu YJ, Malisan F, de Bouteiller O (1996). Within germinal centers, isotype switching of immunoglobulin genes occurs after the onset of somatic mutation. *Immunity*.

[7] Revy P, Muto T, Levy Y (2000). Activation-induced cytidine deaminase (AID) deficiency causes the autosomal recessive form of the Hyper-IgM syndrome (HIGM2). *Cell*.

[8] Muramatsu M, Kinoshita K, Fagarasan S, Yamada S, Shinkai Y, Honjo T (2000). Class switch recombination and hypermutation require activation-induced cytidine deaminase (AID), a potential RNA editing enzyme. *Cell*.

[9] Peled JU, Kuang FL, Iglesias-Ussel MD (2008). The biochemistry of somatic hypermutation. *Annual Review of Immunology*.

[10] Vercelli D (2001). Immunoglobulin E and its regulators. *Current Opinion in Allergy & Clinical Immunology*.

[11] Geha RS, Jabara HH, Brodeur SR (2003). The regulation of immunoglobulin E class-switch recombination. *Nature Reviews Immunology*.

[12] Dedeoglu F, Horwitz B, Chaudhuri J, Alt FW, Geha RS (2004). Induction of activation-induced cytidine deaminase gene expression by IL-4 and CD40 ligation is dependent on STAT6 and NF*κ*B. *International Immunology*.

[13] Messner B, Stütz AM, Albrecht B, Peiritsch S, Woisetschlager M (1997). Cooperation of binding sites for STAT6 and NF*κ*B/rel in the IL-4-induced up-regulation of the human IgE germline promoter. *The Journal of Immunology*.

[14] Cui T, Wang L, Wu J, Hu L, Xie J (2003). Polymorphisms of IL-4, IL-4R*α*, and AICDA genes in adult allergic asthma. *Journal of Huazhong University of Science and Technology*.

[15] Cui TP, Wang L, Jiang WC, Hu LH, Wu JM (2003). Correlation between activation-induced cytidine deaminase gene polymorphism and atopic asthma and plasma IgE in adult. *Chinese Journal of Cellular and Molecular Immunology*.

[16] Noguchi E, Shibasaki M, Inudou M (2001). Association between a new polymorphism in the activation-induced cytidine deaminase gene and atopic asthma and the regulation of total serum IgE levels. *Journal of Allergy and Clinical Immunology*.

[17] Shao C, Suzuki Y, Kamada F (2004). Linkage and association of childhood asthma with the chromosome 12 genes. *Journal of Human Genetics*.

[18] Isidoro-García M, Roa-Gómez S, Davila I, Lorente F, Gonzalez-Sarmiento R (2003). Lack of association between the 7888 C/T polymorphism in the *AID* gene and atopy in a Spanish population. *Journal of Allergy and Clinical Immunology*.

[19] Durandy A, Taubenheim N, Peron S, Fischer A (2007). Pathophysiology of B-cell intrinsic immunoglobulin class switch recombination deficiencies. *Advances in Immunology*.

[20] Imai K, Zhu Y, Revy P (2005). Analysis of class switch recombination and somatic hypermutation in patients affected with autosomal dominant hyper-IgM syndrome type 2. *Clinical Immunology*.

[21] Barreto V, Reina-San-Martin B, Ramiro AR, McBride KM, Nussenzweig MC (2003). C-terminal deletion of AID uncouples class switch recombination from somatic hypermutation and gene conversion. *Molecular Cell*.

[22] Ta V-T, Nagaoka H, Catalan N (2003). AID mutant analyses indicate requirement for class-switch-specific cofactors. *Nature Immunology*.

[23] Shinkura R, Ito S, Begum NA (2004). Separate domains of AID are required for somatic hypermutation and class-switch recombination. *Nature Immunology*.

[24] Levy DA, Chen J (1970). Healthy IgE-deficient person. *The New England Journal of Medicine*.

[25] Ammann AJ, Hong R, Good RA (1970). Healthy IgE-deficient person. *The New England Journal of Medicine*.

[26] Waldmann TA, Polmar SH, Balestra ST, Jost MC, Bruce RM, Terry WD (1972). Immunoglobulin E in immunologic deficiency diseases. II. Serum IgE concentration of patients with acquired hypogammaglobulinemia, thymoma and hypogammaglobulinemia, myotonic dystrophy, intestinal lymphangiectasia and Wiskott-Aldrich syndrome. *The Journal of Immunology*.

[27] Polmar SH, Waldmann TA, Balestra ST, Jost MC, Terry WD (1972). Immunoglobulin E in immunologic deficiency diseases. I. Relation of IgE and IgA to respiratory tract disease in isolated IgE deficiency, IgA deficiency, and ataxia telangiectasia. *The Journal of Clinical Investigation*.

[28] Schoettler JJ, Schleissner LA, Heiner DC (1989). Familial IgE deficiency associated with sinopulmonary disease. *Chest*.

[29] Smith JK, Krishnaswamy GH, Dykes R, Reynolds S, Berk SL (1997). Clinical manifestations of IgE hypogammaglobulinemia. *Annals of Allergy, Asthma & Immunology*.

[30] (1993). Position paper: allergen standardization and skin tests. The European Academy of Allergology and Clinical Immunology. *Allergy*.

[31] Sherry ST, Ward M-H, Kholodov M (2001). dbSNP: the NCBI database of genetic variation. *Nucleic Acids Research*.

[32] Levy Y, Nakum A, Segal N, Monselise Y, Danon YL (2005). The association of selective IgA deficiency and IgE hypogammaglobulinemia. *Allergy*.

[33] Rondón C, Romero JJ, López S (2007). Local IgE production and positive nasal provocation test in patients with persistent nonallergic rhinitis. *Journal of Allergy and Clinical Immunology*.

[34] Rondón C, Doña I, López S (2008). Seasonal idiopathic rhinitis with local inflammatory response and specific IgE in absence of systemic response. *Allergy*.

[35] Gulino AV, Notarangelo LD (2003). Hyper IgM syndromes. *Current Opinion in Rheumatology*.

[36] Vercelli D (2005). Genetic regulation of IgE responses: achilles and the tortoise. *Journal of Allergy and Clinical Immunology*.

